# A Declining CD4 Count and Diagnosis of HIV-Associated Hodgkin Lymphoma: Do Prior Clinical Symptoms and Laboratory Abnormalities Aid Diagnosis?

**DOI:** 10.1371/journal.pone.0087442

**Published:** 2014-02-04

**Authors:** Ravindra K. Gupta, Michael Marks, Simon G. Edwards, Katie Smith, Katie Fletcher, Siow-Ming Lee, Alan Ramsay, Andrew J. Copas, Robert F. Miller

**Affiliations:** 1 Division of Infection and Immunity, University College London, London, United Kingdom; 2 University College London Hospitals’ NHS Foundation Trust, London, United Kingdom; 3 Clinical Research Department, Faculty of Infectious and Tropical Diseases, London School of Hygiene and Tropical Medicine, London, United Kingdom; 4 Mortimer Market Centre, Camden Provider Services, Central and North West London NHS Foundation Trust, London, United Kingdom; 5 Institute of Epidemiology and Healthcare, University College London, London, United Kingdom; The Hospital for Sick Children and The University of Toronto, Canada

## Abstract

**Background:**

The incidence of Hodgkin lymphoma (HL) among HIV-infected individuals remains unchanged since the introduction of combination antiretroviral therapy (cART). Recent epidemiological data suggest that CD4 count decline over a year is associated with subsequent diagnosis of HL. In an era of economic austerity monitoring the efficacy of cART by CD4 counts may no longer be required where CD4 count>350 cells/µl and viral load is suppressed (<50 copies/ml).

**Methods:**

We sought to establish among our HIV outpatient cohort whether a CD4 count decline prior to diagnosis of HL, whether any decline was greater than in patients without the diagnosis, and also whether other clinical or biochemical indices were reliably associated with the diagnosis.

**Results:**

Twenty-nine patients with a diagnosis of HL were identified. Among 15 individuals on cART with viral load <50 copies/ml the change in CD4 over 12 months preceding diagnosis of HL was −82 cells/µl (95% CI −163 to −3; p = 0.04). Among 18 matched controls the mean change was +5 cells/µl, 95% CI −70 to 80, p = 0.89). The decline in CD4 over the previous 6–12 months was somewhat greater in cases than controls (mean difference in change −55 cells/µl, 95% CI −151 to 39; p = 0.25). In 26 (90%) patients B symptoms had been present for a median of three months (range one–12) before diagnosis of HL.

**Conclusions:**

The CD4 count decline in the 12 months prior to diagnosis of Hodgkin lymphoma among HIV-infected individuals with VL<50 copies/ml on cART was not significantly different from that seen in other fully virologically suppressed individuals in receipt of cART and who did not develop HL. All those who developed HL had B symptoms and/or new palpable lymphadenopathy, suggesting that CD4 count monitoring if performed less frequently, or not at all, among those virologically suppressed individuals with CD4 counts >350 may not have delayed diagnosis.

## Introduction

While the incidence of AIDS-defining malignancies such as Kaposi sarcoma and non-Hodgkin lymphoma has fallen since the introduction of highly active antiretroviral therapy (HAART) [1], [2], the incidence of Hodgkin lymphoma has not decreased, and remains elevated in HIV infected individuals [3]–[7]. Although Hodgkin lymphoma in the context of HIV has been associated with a more aggressive clinical course and a preponderance for more aggressive histological subtypes [8], [9], the prognosis of Hodgkin lymphoma has improved and response rates are similar to those in the HIV uninfected population [10]–[12].

An association between a falling CD4 cell count, not explained by virological failure of HAART, and a diagnosis of Hodgkin lymphoma has been described by the COHERE collaboration [13]. The authors of that study proposed monitoring of CD4 counts and that a declining CD4 count might alert clinicians to the possibility of Hodgkin lymphoma. However, a transient CD4 decline to <350 cells/mm^3^ among virologically suppressed patients is common [2], [14] and, given the low incidence of Hodgkin lymphoma among HIV-infected patients in Northern Europe, it is possible that a transient decline in CD4 count may trigger clinicians to undertake unnecessary investigations and follow up.

We reviewed the CD4 dynamics, HAART history and clinical features in virologically suppressed patients with HIV-associated Hodgkin lymphoma and among matched controls to assess whether a CD4 count decline in virologically suppressed patients was indicative of development of Hodgkin lymphoma, and to identify if such a decline, if present, was associated with any clinical or laboratory markers suggestive of Hodgkin lymphoma.

## Materials and Methods

We undertook a retrospective cohort study of all HIV-associated Hodgkin lymphoma diagnoses at our University-affiliated hospital-based service in London, between 1996 and 2012. Mortimer Market Centre and University College London Hospitals provide HIV care to over 4,000 individuals in North central London, United Kingdom (UK). Patients were managed according to UK National Guidelines, with clinical review and CD4 monitoring every three to six months [15]. CD4 lymphocyte count monitoring was done in laboratory facilities (accredited to UK national standards) at University College London Hospitals. The same assay was used throughout the study and the laboratory is accredited for CD4 measurements with QC performed regularly. All CD4 counts are drawn and processed in the same working day. Antiretroviral regimens were prescribed according to UK national guidelines [15]. In the United Kingdom retrospectively obtained anonymised NHS data used for audit and service improvement purposes does not require ethical approval.

For each individual with Hodgkin lymphoma data were extracted from paper case note review and interrogation of electronic patient records, including viral load, antecedent CD4 dynamics (absolute CD4 count) prior to diagnosis of Hodgkin lymphoma, as well as clinical and laboratory parameters. All patients in the cohort who received HAART did so with two nucleoside reverse transcriptase inhibitors and one non-nucleoside reverse transcriptase inhibitor, a boosted protease inhibitor, or an integrase inhibitor. Individuals with a fully suppressed viral load (<50 copies/ml) and in receipt of HAART at diagnosis of Hodgkin lymphoma were matched with controls who were attending the outpatient clinic at Mortimer Market Centre, over the same time interval and who were also in receipt of HAART and had a fully suppressed viral load, and who did not subsequently develop Hodgkin lymphoma. For each of these control patients equivalent data was abstracted from case notes and electronic patient records, relating to a ‘diagnosis date’ close to diagnosis in the matched case and 12 months prior. Controls were matched to cases on the basis of age (±6 months), gender, and CD4 lymphocyte count (±100 cells/mm^3^) at the earliest date (6, 9 or 12 months) prior to the ‘diagnosis’ date for which there was a value available for both cases and controls. Where possible two controls were matched to each case.

The Fisher’s exact test (two-tailed) was used to compare proportions; the matched pairs Students’ T-test was used to compare CD4 counts at baseline and at diagnosis for cases and controls separately. These tests were carried out in GraphPad Prism v4.01 (GraphPad Software Inc, San Diego, USA). To compare the change in CD4 over time in cases with their matched controls, linear regression with the change in CD4 as outcome variable and case or control status as predictor was used, and generalised estimating equations in Stata 12 (StataCorp LP, College Station, USA) provided robust standard errors that recognised the matching. Analysis of change in CD4 count in cases was based on the change from the earliest date (6, 9, or 12 months) prior to diagnosis for which the CD4 count was measured to diagnosis, and for comparison of cases to controls change from the earliest date where the CD4 was measured for both case and matched control(s) was analysed. A p-value of <0.05 was considered significant.

## Results

### Demographics and Presentation

Between January1996 and May 2012 a histologically-confirmed diagnosis of Hodgkin lymphoma was made in 29 HIV-infected patients: 27 (93%) were male and six (21%) were Black African. Eighteen were men who have sex with men and two were intravenous drug users. Three patients were co-infected with Hepatitis B and two were co-infected with Hepatitis C. Their median age was 41 years (range 26–61) and median time from diagnosis of HIV infection to diagnosis of Hodgkin lymphoma was 67 months (range 0–320). No patient received interferon-γ and none started co-trimoxazole. Fifteen patients were receiving HAART and were fully suppressed (VL<50), fourteen patients were not in receipt of HAART and had detectable viral loads. Fifteen patients, fully suppressed (VL<50) were identified. These cases were matched to 18 control patients (also virologically suppressed, who presented at the same time, were on HAART and who did not develop HL).

At diagnosis of Hodgkin lymphoma, eighteen individuals had stage IVB, two IVA, four IIIB, three IIB, one 1A and one stage 1B disease. Seventeen (59%) had bone marrow involvement; one patient did not undergo bone marrow examination, and this was the only site of positive histology in 7/29 (24%) episodes of Hodgkin lymphoma. At diagnosis of Hodgkin lymphoma 13 patients had a ^18^FDG PET-CT scan. Three patients with histologically-confirmed bone marrow involvement had ^18^FDG PET-CT scans suggestive of bone marrow infiltration. Of the other ten patients with no evidence of bone marrow involvement on ^18^FDG PET-CT, five had Hodgkin lymphoma on bone marrow biopsy.

Among patients with advanced disease (Stage III/IV, n = 24) the median lymphoma prognostic score (IPS) [16] of the group at diagnosis was 3 (IQR 2–4), and 67% had a score of ≥3, similar to that described in other UK centres [12]. Among the 12 virologically–suppressed patients with advanced disease the median IPS was 3 (IQR 2.75–4); in three patients IPS <3 and in nine IPS ≥3. All but two patients had a complete response: of the two patients who relapsed, one had a score of 5 and the other a score of 2. The latter patient died of Hodgkin lymphoma while the former achieved a good outcome.

### Dynamics of CD4 Counts Preceding Diagnosis of Hodgkin Lymphoma

Seventeen (59%) individuals had started HAART prior to diagnosis of Hodgkin lymphoma (median duration was 3.5 years, IQR 1.5–9.5 years). Fifteen (52%) individuals had a plasma HIV RNA <50 copies/ml at diagnosis of Hodgkin lymphoma; HIV RNA was >50 copies/ml (median 50,500 copies/ml, IQR 29,375–177,100 copies/ml) in 12; plasma HIV RNA was not recorded in two individuals. Median CD4 count at diagnosis of Hodgkin lymphoma was 270 cells/µl (IQR 153–368). Median CD4 count in those with plasma HIV RNA <50 copies/ml was 320 cells/µl (IQR 188–368), and 240 cells/µl (IQR 150–330) in those with plasma HIV RNA >50 copies/ml.

**Figure 1:**
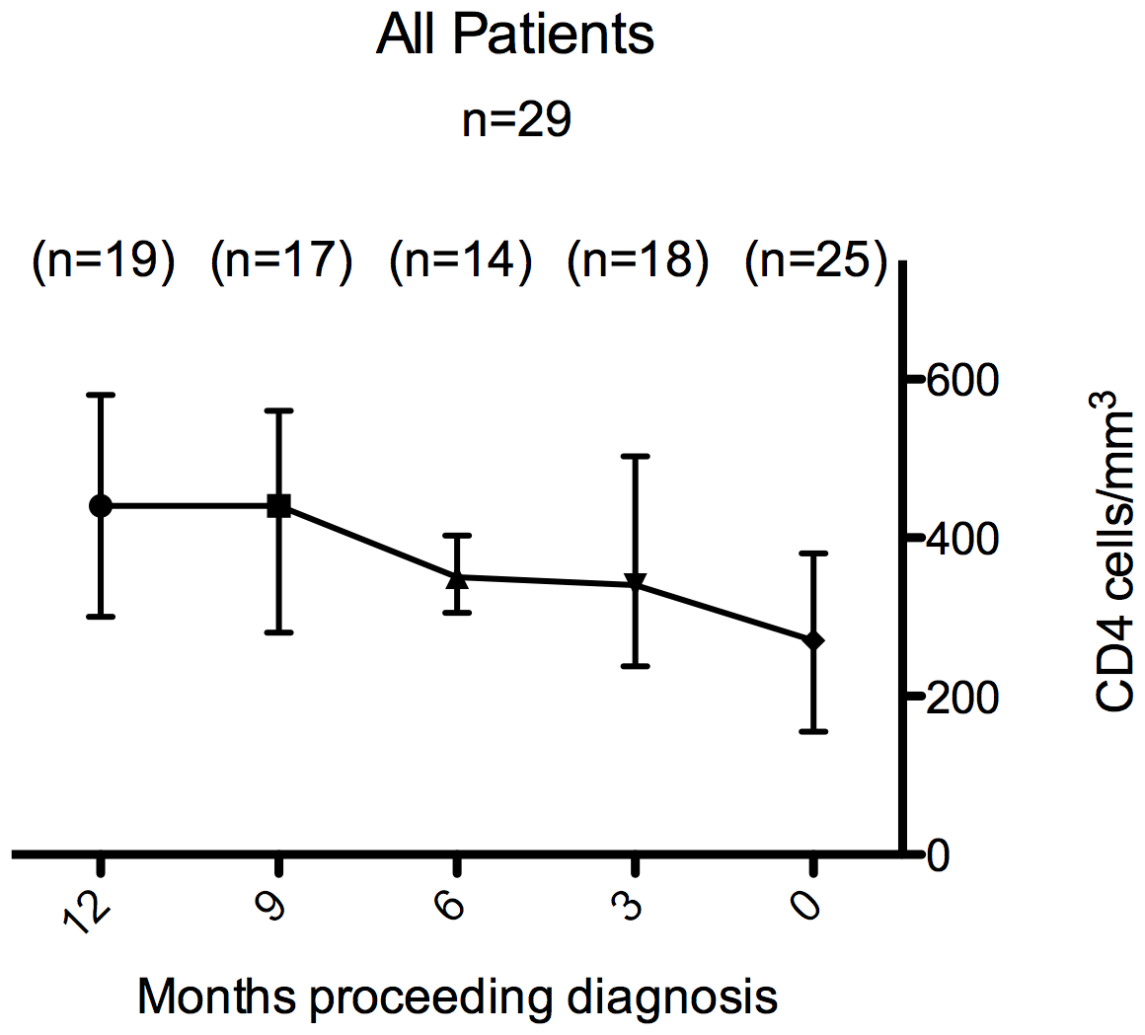
Change in CD4 count in the twelve months preceding diagnosis of Hodgkin Lymphoma amongst 29 patients studied. Data points are median CD4 in the cohort at each time point and hairs represent interquartile range. Numbers in parentheses represent the number of observations at each time point.

**Figure 2:**
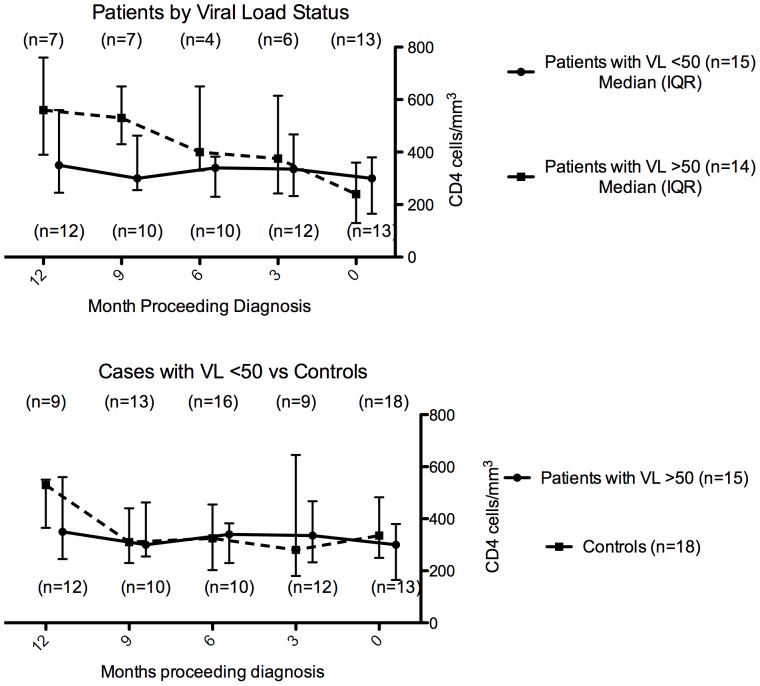
(A) Change in CD4 count in the twelve months preceding diagnosis of Hodgkin Lymphoma amongst patients with and without a fully suppressed viral load. (B) Change in CD4 count in the twelve months preceding diagnosis of Hodgkin Lymphoma in cases with a fully suppressed viral load as compared to CD4 counts in matched HIV positive control individuals without HL over a similar time period. Data points are median CD4 in the cohort at each time point and hairs represent interquartile range. Numbers in parentheses represent the number of observations at each time point.

There was a significant fall in CD4 count over the 6–12 months prior to diagnosis of Hodgkin lymphoma (mean change −128 cells/µl, 95% CI −194 to −64; p = 0.001), Figure 1. Among 17 patients receiving HAART at diagnosis of Hodgkin lymphoma CD4 counts also declined, (mean change = −89 cells/µl, 95% CI −153 to −2; p = 0.04). When analysis was restricted to the 15 patients with consistent plasma HIV RNA <50 copies/ml in the 12 months preceding diagnosis of Hodgkin lymphoma results were similar; mean change in CD4 count was −82 cells/mm^3^ (95% CI −163 to −3; p = 0.04), Figure 2. The median number of CD4 count measurements per individual was 4 (IQR 2–5). There was little change over time in CD4 counts amongst the 18 controls (mean change = 5 cells/µl, 95% CI −70 to 80, p = 0.89). The decline in CD4 over the previous 6–12 months was somewhat greater in cases than controls (mean difference in change −55 cells/µl, 95% CI −151 to 39; p = 0.25), Figure 2. Among those with suppressed viral load (VL<50), only 7/15 were lymphopenic at diagnosis of Hodgkin lymphoma. Six of the seven virologically suppressed patients with lymphopenia had stage IVB disease and one had stage IIIB disease. The median IPS in these patients was 3 (IQR 2.5–3.5). Among the eight patients who were virologically suppressed but not lymphopenic three had Stage IVB, two had stage IVA, two had stage IIB and one had stage 1A disease. The median IPS in the non-lymphopenic patients with advanced disease was 4 (IQR 3–4). Lymphopenia at diagnosis of Hodgkin lymphoma was not associated with stage of disease (p = 0.57) or IPS (p = 0.61). We found a trend towards association between lymphopenia at diagnosis of Hodgkin lymphoma and CD4 decline in the 12 months prior to diagnosis (p = 0.11). The mean change in CD4 count in the seven lymphopenic patients was −142 cells/µl (95% CI −244 to −40), and −30 cells/µl (95% CI −124 to +64) in the eight non-lymphopenic patients, However, the difference did not reach statistical significance (p = 0.1393). The CD4 decline was normally distributed (data not shown) in the subgroups considered.

### Clinical and Biochemical Indices in Patients with Hodgkin Lymphoma

In 26 (90%) patients B symptoms had been present for a median of 3 months (range 1–12). Three patients did not have B symptoms (all were on HAART); one had Stage IA disease and symptoms of less than one month duration, one had Stage IVA disease and no change in CD4 count, and one had a fall in CD4 lymphocyte count without B symptoms and multiple hematological and biochemical abnormalities prior to diagnosis: all three had new palpable peripheral lymphadenopathy. No controls developed B symptoms or new palpable lymphadenopathy.

Nineteen patients had anaemia and or thrombocytopenia prior to diagnosis of Hodgkin lymphoma. Cytopenias at diagnosis were not associated with bone marrow involvement (p = 0.47). One of the controls (6%) developed new cytopenias, which were transient.

## Discussion

The incidence of Hodgkin lymphoma has not fallen in HIV infected patients in the era of HAART [3]–[5]. One of the main challenges for clinicians is to rapidly make a diagnosis of Hodgkin lymphoma among patients with non-specific symptoms such as fever, sweats and weight loss. In the COHERE study patients with Hodgkin lymphoma and who were in receipt of HAART lost 98 CD4 lymphocytes (95% CI, −159 to −36 cells) in the year preceding diagnosis whilst maintaining viral suppression on HAART [13]. In the present study we saw a similar decline in CD4 count over this period among patients receiving HAART and who were virologically suppressed. No significant change in CD4 count was observed in matched controls. Notably no individuals received interferon-γ nor started co-trimoxazole during the study period. Although a minority received didanosine or zidovudine these agents were not started during the year preceding the diagnosis of Hodgkin lymphoma. Therefore the observed fall in CD4 count in patients with Hodgkin lymphoma cannot be ascribed to drugs that might have suppressed their CD4 counts.

Data from high income settings suggest that CD4 monitoring is not required in addition to routine viral load measurement when CD4 counts are above 350 cells/µl and viral suppression has been achieved [14], [17]. With increasing focus on health care efficiency, such findings will lead to far fewer lymphocyte subset assays being performed. Given the results from the COHERE study (mean decline in CD4 count in the year before diagnosis of Hodgkin lymphoma in virologically suppressed patients reciving HAART was 98 cells/µl) [13] and other recent data from Italy showing a mean CD4 count decline of 99 cells/µl in the year before diagnosis of Hodgkin lymphoma, compared with a mean increase of 37 cells/µl in controls [7], it might be inferred that an opportunity will be missed to make the diagnosis. However, our data suggest that stopping routine monitoring of CD4 counts would not be expected to result in diagnosis of Hodgkin lymphoma being missed or delayed if regular clinical-follow up is maintained, and clinicians are aware of the importance of monitoring for B symptoms, new palpable peripheral lymphadenopathy or abnormal blood biochemistry/cytopenias. New palpable lymphadenopathy and B symptoms would have captured all cases of Hodgkin lymphoma in our cohort. The majority of our patients had bone marrow involvement and of note a substantial minority had isolated bone marrow disease, highlighting the importance of this examination as part of a work up for suspected Hodgkin lymphoma. It is now our practice to educate clinicians and patients to be alert for the onset and significance of B symptoms and for the development of new lymphadenopathy.

Our study is limited by the relatively small sample size and its retrospective nature. Larger prospective studies of patients with suppressed viral loads are indicated to evaluate the utility of monitoring for classical symptoms and signs of lymphoma (B symptoms, cytopenias, lymphadenopathy) compared with the utility of CD4 count monitoring for early detection of patients with Hodgkin lymphoma.

In summary, this study showed that there was a CD4 count decline in the 12 months prior to diagnosis of Hodgkin lymphoma among HIV-infected individuals who were fully virologically suppressed and in receipt of HAART. All those who developed Hodgkin lymphoma had B symptoms and/or new palpable lymphadenopathy.

## References

[1] Engels EA, Pfeiffer RM, Goedert JJ, Virgo P, McNeel TS, et al (2006) Trends in cancer risk among people with AIDS in the United States 1980–2002. AIDS 20: 1645–1654. 16868446 10.1097/01.aids.0000238411.75324.59

[2] Biggar RJ, Chaturvedi AK, Goedert JJ, Engels EA, Study HACM (2007) AIDS-related cancer and severity of immunosuppression in persons with AIDS. J Natl Cancer Inst 99: 962–972. 17565153 10.1093/jnci/djm010

[3] Clifford GM, Rickenbach M, Lise M, Dal Maso L, Battegay M, et al (2009) Hodgkin lymphoma in the Swiss HIV Cohort Study. Blood 113: 5737–5742. 19336755 10.1182/blood-2009-02-204172

[4] Franceschi S, Lise M, Clifford GM, Rickenbach M, Levi F, et al (2010) Changing patterns of cancer incidence in the early- and late-HAART periods: the Swiss HIV Cohort Study. Br J Cancer 103: 416–422. 20588274 10.1038/sj.bjc.6605756 PMC2920013

[5] Dal Maso L, Polesel J, Serraino D, Lise M, Piselli P, et al (2009) Pattern of cancer risk in persons with AIDS in Italy in the HAART era. Br J Cancer 100: 840–847. 19223894 10.1038/sj.bjc.6604923 PMC2653754

[6] Biggar RJ, Jaffe ES, Goedert JJ, Chaturvedi A, Pfeiffer R, et al (2006) Hodgkin lymphoma and immunodeficiency in persons with HIV/AIDS. Blood 108: 3786–3791. 16917006 10.1182/blood-2006-05-024109 PMC1895473

[7] Gotti D, Danesi M, Calabresi A, Ferraresi A, Albini L, et al (2013) Clinical characteristics, incidence, and risk factors of HIV-related Hodgkin lymphoma in the era of combination antiretroviral therapy. AIDS Patient Care STDS 27: 259–265. 23600703 10.1089/apc.2012.0424

[8] Grogg KL, Miller RF, Dogan A (2007) HIV infection and lymphoma. J Clin Pathol 60: 1365–1372. 18042692 10.1136/jcp.2007.051953 PMC2095580

[9] Tirelli U, Errante D, Dolcetti R, Gloghini A, Serraino D, et al (1995) Hodgkin's disease and human immunodeficiency virus infection: clinicopathologic and virologic features of 114 patients from the Italian Cooperative Group on AIDS and Tumors. J Clin Oncol 13: 1758–1767. 7541452 10.1200/JCO.1995.13.7.1758

[10] Gerard L, Galicier L, Boulanger E, Quint L, Lebrette MG, et al (2003) Improved survival in HIV-related Hodgkin's lymphoma since the introduction of highly active antiretroviral therapy. Aids 17: 81–87. 12478072 10.1097/00002030-200301030-00011

[11] Hoffmann C, Chow KU, Wolf E, Faetkenheuer G, Stellbrink HJ, et al (2004) Strong impact of highly active antiretroviral therapy on survival in patients with human immunodeficiency virus-associated Hodgkin's disease. Br J Haematol 125: 455–462. 15142115 10.1111/j.1365-2141.2004.04934.x

[12] Montoto S, Shaw K, Okosun J, Gandhi S, Fields P, et al (2012) HIV Status Does Not Influence Outcome in Patients With Classical Hodgkin Lymphoma Treated With Chemotherapy Using Doxorubicin, Bleomycin, Vinblastine, and Dacarbazine in the Highly Active Antiretroviral Therapy Era. J Clin Oncol 30: 4111–4116. 23045581 10.1200/JCO.2011.41.4193 PMC5320889

[13] Bohlius J, Schmidlin K, Boue F, Fatkenheuer G, May M, et al (2011) HIV-1-related Hodgkin lymphoma in the era of combination antiretroviral therapy: incidence and evolution of CD4(+) T-cell lymphocytes. Blood 117: 6100–6108. 21368291 10.1182/blood-2010-08-301531

[14] Whitlock GG, Ahmed N, Benn P, Edwards S, Waters L (2013) Stop routine CD4 monitoring in HIV-infected patients with fully suppressed virus and CD4> = 350 cells/ml. Clin Infect Dis 57: 327–328. 23537910 10.1093/cid/cit203

[15] BHIVA (2012) British HIV Association guidelines for the treatment of HIV-1-positive adults with antiretroviral therapy 2012. HIV Med 13 (Suppl. 2)1–85. 10.1111/j.1468-1293.2012.01029.x 22830364

[16] Hasenclever D, Diehl V (1998) A prognostic score for advanced Hodgkin's disease. International Prognostic Factors Project on Advanced Hodgkin's Disease. N Engl J Med 339: 1506–1514. 9819449 10.1056/NEJM199811193392104

[17] Gale HB, Gitterman SR, Hoffman HJ, Gordin FM, Benator DA, et al (2013) Is frequent CD4+ T-lymphocyte count monitoring necessary for persons with counts > = 300 cells/muL and HIV-1 suppression? Clin Infect Dis 56: 1340–1343. 23315315 10.1093/cid/cit004 PMC3693489

